# Garlic (*Allium sativum*) feature-specific nutrient dosage based on using machine learning models

**DOI:** 10.1371/journal.pone.0268516

**Published:** 2022-05-17

**Authors:** Leandro Hahn, Léon-Étienne Parent, Angela Cristina Paviani, Anderson Luiz Feltrim, Anderson Fernando Wamser, Danilo Eduardo Rozane, Marcos Matos Ender, Douglas Luiz Grando, Jean Michel Moura-Bueno, Gustavo Brunetto

**Affiliations:** 1 Caçador Experimental Station, Agricultural Research and Rural Extension Agency of Santa Catarina (Epagri), Caçador, Santa Catarina, Brazil; 2 Department of Soils and Agrifood Engineering, Laval University, Québec, Canada; 3 Department of Soil, Federal University of Santa Maria, Santa Maria, Rio Grande do Sul, Brazil; 4 Executive Secretariat for the Environment, Government Administrative Center, Florianópolis, Santa Catarina, Brazil; 5 State University of Paulista “Julio Mesquita Filho”, Campus Registro, São Paulo, Brazil; 6 University of Alto Vale do Rio do Peixe, Caçador, Santa Catarina, Brazil; Wageningen University, NETHERLANDS

## Abstract

Brazil presents large yield gaps in garlic crops partly due to nutrient mismanagement at local scale. Machine learning (ML) provides powerful tools to handle numerous combinations of yield-impacting factors that help reducing the number of assumptions about nutrient management. The aim of the current study is to customize fertilizer recommendations to reach high garlic marketable yield at local scale in a pilot study. Thus, collected 15 nitrogen (N), 24 phosphorus (P), and 27 potassium (K) field experiments conducted during the 2015 to 2017 period in Santa Catarina state, Brazil. In addition, 61 growers’ observational data were collected in the same region in 2018 and 2019. The data set was split into 979 experimental and observational data for model calibration and into 45 experimental data (2016) to test ML models and compare the results to state recommendations. Random Forest (RF) was the most accurate ML to predict marketable yield after cropping system (cultivar, preceding crops), climatic indices, soil test and fertilization were included features as predictor (R^2^ = 0.886). Random Forest remained the most accurate ML model (R^2^ = 0.882) after excluding cultivar and climatic features from the prediction-making process. The model suggested the application of 200 kg N ha^-1^ to reach maximum marketable yield in a test site in comparison to the 300 kg N ha^-1^ set as state recommendation. P and K fertilization also seemed to be excessive, and it highlights the great potential to reduce production costs and environmental footprint without agronomic loss. Garlic root colonization by arbuscular mycorrhizal fungi likely contributed to P and K uptake. Well-documented data sets and machine learning models could support technology transfer, reduce costs with fertilizers and yield gaps, and sustain the Brazilian garlic production.

## Introduction

Garlic (*Allium sativum* L.) is an appreciated human food presenting high carbohydrate, phosphoric and sulfuric acids, proteins and mineral salts contents, as well as antioxidant, antimicrobial, anti-inflammatory, anti-thrombosis, anti-cancer and anti-atherosclerosis properties [1–3]. The Brazilian garlic production peaked at 132,000 tons per year from 2016 to 2019 [4]; however, it only covered 33% of its domestic demand. Such a considerable gap between production and demand [2] poses serious challenge to the development of a sustainable garlic industry in Brazil [5].

Garlic yield in Brazil reaches 11 Mg ha^-1^, on average; it ranged from 4 to 18 Mg ha^-1^ in recent crops [6]. Temperature, rainfall and photoperiod are the variables mostly influencing bulb yield at regional scale [7, 8]. There is great potential to increase garlic yield through genetic enhancement programs [5, 9, 10], optimum plant density [11], pest management [12], irrigation [13] and site-specific fertilization [2, 6, 14]. Fertilization of annual crops such as garlic is generally guided by field experiments, soil testing procedures [15] and by the anticipated effect of both preceding crops (biomass N) and soil organic matter [16, 17].

Soil tests are more closely correlated to relative yield than to absolute yield; therefore, agronomy traditionally uses relative yield, since it assumes that all factors are at equal or optimum levels, except for those that actually vary [18]. Relative yield (RY) is calculated by dividing the yield in the control treatment by the maximum yield at each site [19], or as response ratio (yield in treatment divided by yield in control) weighted by the variance recorded for each experiment [20]. RY models allowed setting the soil test for P and K fertility classes in order to elaborate ‘universal’ state fertilizer recommendations [19, 21, 22]. However, nutrient dose at optimum economic yield cannot be measured through RY. Actually, it is not common to see all elements at optimum level, or near to it, in field experiments [23]. Growers tend to over-fertilize crops to avoid yield loss by using ‘universal’ models [24–26]. On the other hand, growers tend to compare defective and successful crop performance in their neighborhood in order to identify the factors most often limiting plant growth [23].

Garlic economic viability is strongly affected by high costs for fertilizers, mainly for nitrogen [27]. Excessive N fertilization is often seen as capable of guaranteeing high crop yield [14, 24, 25]. Phosphorus, in its turn, is often applied in excessive amounts in Brazil, due to its high soil phosphorus-sorption capacity that assumingly limits plant access to soil reserves [26, 28]. Empirical coefficients have been proposed to account for P sorption capacity [28], without taking into consideration the plants’ ability to acquire phosphorus from soil and fertilizers [26].

Physical crop models measure crop response to N and water stress based on empirical coefficients and datasets, at regional scale [29–31]. Meta-analysis of N trials [2], Maximum Return to Nitrogen [32] and machine learning-compositional models [33] could also support decision-making on fertilization procedures. Former soil test models could be gradually replaced by customized fertilizer recommendation models given the increasing access to big data [34–36]. Machine learning models can handle site-specific growth-impacting factors [32–40].

We hypothesized that machine learning models can provide fertilizer prescriptions that are quite different from recommendations, as proof of concept in a pilot study, at local scale. Our aim was to customize fertilizer recommendations to reach high garlic marketable yield at local scale in a pilot study carried out in Southern Brazil.

## Material and methods

### Experimental setup

This pilot project was conducted in *Fraiburgo* County, *Santa Catarina* State, Brazil (-27.04 S, -50.83W). The authorization to collect samples was granted during a meeting with producers, who allowed setting up the experiments and collecting samples. The meeting was organized by Santa Catarina Association of Garlic Producers (ACAPA); local producers willing to collaborate with the research volunteered to it and also authorized the experiments. All producers participating in the research are members of ACAPA, which is the local authority that approved the research.

Climate in the investigated region is temperate and humid, with mild Summer; according to *Köppen’s* classification, it is classified as Cfb [40]. Landscape was moderately flat to slightly undulated. Soils were classified as Typic Hapludox [41]. Soil cation exchange capacity (CEC) at soil surface (0–20 cm) ranged from 13 to 23 cmol_c_ kg^-1^. Base saturation of CEC, computed as the sum of exchangeable cations, divided by CEC, ranged from 20% and 94%.

We collected edaphic, climatic and managerial data from field fertilizer experiments and growers. Plots comprised three double-line planting beds (5 m in length) [14]. Seeds were obtained from virus-free meristem culture of noble group cultivars ‘Chonan’, ‘Ito’, ‘Quitéria’, ‘Roxo Caxiense’ and ‘São Valentin’ (Table 1), which account for the greatest yield potential in Brazil. These cultivars have purple bulbs and require several days for bulb formation [5, 7]. Seed cloves were vernalized at 2–5°C, for 20–30 days, before planting. Planting density was 45 seed cloves m^-2^. Preceding crops encompassed maize (*Zea mays*), soybean (*Glycine max*), bean (*Phaseolus vulgaris*) or garlic (*Allium sativum*). Other cultural practices comprised commonly used in the region. Garlic crops were sprinkler-irrigated.

**Table 1:** Yearly distribution of experimental and observational data in the Brazilian garlic data set.

Year	2015			2016			2017	2018	2019	Total
	N	P	K	N	P	K	N	Growers		
Number of trials	5	14	15	6	11	11	4	1	2	69
Number of observations	71	210	225	88	150	170	48	28	33	1023

Fertilizer trials were conducted with nitrogen (N), phosphorus (P) and potassium (K). There were 34 fertilizer trials in 2015 (5 N, 14 P and 15 K); 28, in 2016 (6 N, 10 P and 12 K); and 4, in 2017 (4 N) (Table 1). There were three additional observational ‘Chonan’ sites located in one farm in 2018, and in two farms in 2019. There were 1,023 observations, in total, including data about the 61 growers, which were collected in 2018–2019. History of preceding crops and fertilization gathered during the 1,023 observations are presented in S1 Table in S1 File.

In order to compare data sets, ML and broken curve regression models were run across 78 experiments in USA to measure corn (*Zea Mays*) response to P addition [42] and across 370 experiments in Canada [35] to model potato (*Solanum tuberosum*) response to fertilization (273 field trials, 5,913 lines). A study with 198 fertilizer experiments carried out in Midwest US modeled N response in corn [32]. Data from 1,504 experimental wild blueberry data were collected for 11 years [38]. The size of other data sets to run ML models ranged from 60–240 to nearly 8,000 observations—smaller numbers correspond to more difficulties and higher costs with data gathering [32, 42–50].

Experimental setups followed the randomized block design with three repetitions. Fertilizers were broadcast-applied and incorporated into the 0.20 m layer with the aid of a rotary tiller. The experimental sites received five N doses (0, 100, 200, 300 or 400 kg N ha^-1^) as ammonium nitrate, 1/3 of them were applied in the rows at planting time; 1/3 was used as top-dressing, 30 days after planting; and 1/3 was used as top-dressing at bulb formation time (approximately 95 days after planting, when plants differentiated into bulbs). Five (5) P doses in the form of triple superphosphate were applied on the soil, as well as 5 K doses in the form of potassium chloride—all doses were applied before planting. Sites presenting N variation were subjected to P and K application at equal doses of 175 kg P ha^-1^ and 333 kg K ha^-1^, respectively. Sites presenting P and K content variations were subjected to 3 N applications (100 kg N ha^-1^) during the season–these applications total 300 kg N ha^-1^. Triple superphosphate was used as P source, whereas potassium chloride was used as K source. Application rates were 160 kg N ha^-1^, 680 kg P_2_O_5_ ha^-1^ and 457 kg K_2_O ha^-1^ in the 2018 site; 130 kg N ha^-1^, 300 kg P_2_O_5_ ha^-1^ and 150 kg K_2_O ha^-1^ in the 2019A site; and 180 kg N ha^-1^, 200 kg P_2_O_5_ ha^-1^ and 200 kg K_2_O ha^-1^ in the 2019B site. N, P and K sources and irrigation were the same as described above.

Bulbs were collected in uniform 1m length rows, comprising 3 double plant lines per plot [14]. They were weighed after ⁓40 days of natural drying. Marketable bulbs corresponded to the sum of #2 (< 32 mm ∅), #3 (32–37 mm ∅), #4 (37–42 mm ∅), #5 (42–47 mm ∅), # 6 (47–56 mm ∅) and #7 (> 56 mm ∅) bulbs, which were classified according to [51]. Bulbs showing secondary growth, or damage, were considered nonmarketable. Maximum yield ranged from 7.8 and 13.3 Mg ha^-1^ over the years; it was comparable to yield of 9.2–13.4 Mg ha^-1^ –which was recorded by [52] in the same region.

### Soil analyses

Eight (8) soil subsamples were collected per plot in the 0–0.20 m soil layer, at harvest time. Soil samples were air-dried and ground to lesser than 2-mm in size, before chemical analyses were performed based on [53], as follows: pH 1:1 soil:water volumetric ratio; clay, based on the densimeter method; Mehlich-1 extraction method for P, K, Cu, Zn, and Fe; KCl method for Mn; and hot-water extraction for B. Elements were quantified through plasma-emission spectroscopy (ICP-OES). Total carbon was quantified based on dichromate oxidation (Walkley–Black) and multiplied by 1.724 to find the organic matter content [54]. Clay content was determined based on sedimentation. Cation exchange capacity was computed as the sum of exchangeable cations and total acidity (SMP buffer pH). Values recorded during soil P and K tests applied to the control treatment were selected for modeling purposes. The Brazilian fertilizer recommendation system [55] adjusts P and K recommendations to soil clay content (P) and to cation exchange capacity (K). Variation in soil properties in experimental sites is shown in S2 Table in S1 File.

### Climatic indices

Annual crops’ performance in comparison to that of the perennial ones is little affected by off-season climatic conditions. Only adverse temperatures, or extreme meteorological events, such as the excessive rainfall recorded in 2015, can have impact on crop yield, in places subjected to irrigation. The following meteorological data were collected in the closest [56] meteorological station to study area: daily minimum and maximum temperatures, daily rainfall between planting and harvest time, and growing season duration. All experiments were located within the radius of 50 km away. The meteorological station used for data collection was located within this radius. The yearly distribution of experimental and observational data in the Brazilian garlic data set is shown in S3 Table in S1 File.

Plantation dates went from May 7^th^ to July 26^th,^ depending on site and year. Minimum, median and maximum Julian days for plantation were 152, 180, and 238, respectively. Harvest dates went from November 3^rd^ to December 5^th^. Minimum, median and maximum Julian days for harvest were 306, 322, and 342, respectively. Growing period duration was computed based on the difference between Julian day at planting and Julian day at harvest. Minimum, median and maximum growing days were 102, 143, and 162, respectively. Heat accumulation was measured as cumulated degree-days above critical temperature during the growing season. Critical temperature for cold crops was 5°C [57].

### Statistical analyses

#### Traditional models

Crop yield was associated with N dose in corn or soybean crops used as preceding crops. Crop response to N fertilization could be low when N credits are high preceding crops (soybean). Soil organic matter also provides N credits [55].

RY of P and K was calculated at each experimental site as %*Yield* = 100 × (*Y*_*control*_⁄*Y*_*maximum*_), wherein *Y*_*control*_ is yield in control treatment zero and *Y*_*maximum*_ is maximum yield [19]. Numbers of points are maximized in opposite quadrants in order to compute classification accuracy. Critical soil test separates high from low crop response to P or K addition.

#### Machine learning models

Machine learning models can predict phenomenological outcomes from key features [58]. Random Forest, k-nearest neighbors (KNN), support vector machine (SVM), gradient boosting, stochastic gradient descent (SGD), Adaboost, linear regression and Neural Networks were the ML models available in Orange data mining freeware vs. 3.23. Decision trees were commonly used in plant nutrition studies [35, 39].

Marketable yield, the logistic partition between marketable and non-marketable yield were the target variables. The logistic partition was computed as *ln*(*Yield*_*marketable*_⁄*Yeld*_*rejected*_). Predictor features were set as follows: N, P and K dosage, soil tests, cultivar, cropping system (preceding crop, seeding or planting), and climatic indices (growing season duration, cumulated degree-days, total seasonal rainfall).

Models were calibrated through stratified cross-validation (k = 10). The data set was divided into 979 experimental and observational data for calibration, and into 45 experimental data (2016) to test ML models and compare results to ‘universal’ state recommendations. The training data set excluded one P trial in 2016, one K trial in 2016, and one N trial in 2017, in cultivar ‘Ito’, which was used as testing data set. The most accurate ML regression models were selected by using the training data set. Accuracy was measured as root mean-square error (RMSE), mean absolute error (MAE) and R^2^ statistics. Predictors contribution to model accuracy was assessed by removing predictors and examining any change in model accuracy. Excluded trials were used for model testing. Garlic response predicted by the models were compared to the observed response.

## Results

### Traditional crop-response models

**Fig 1:**
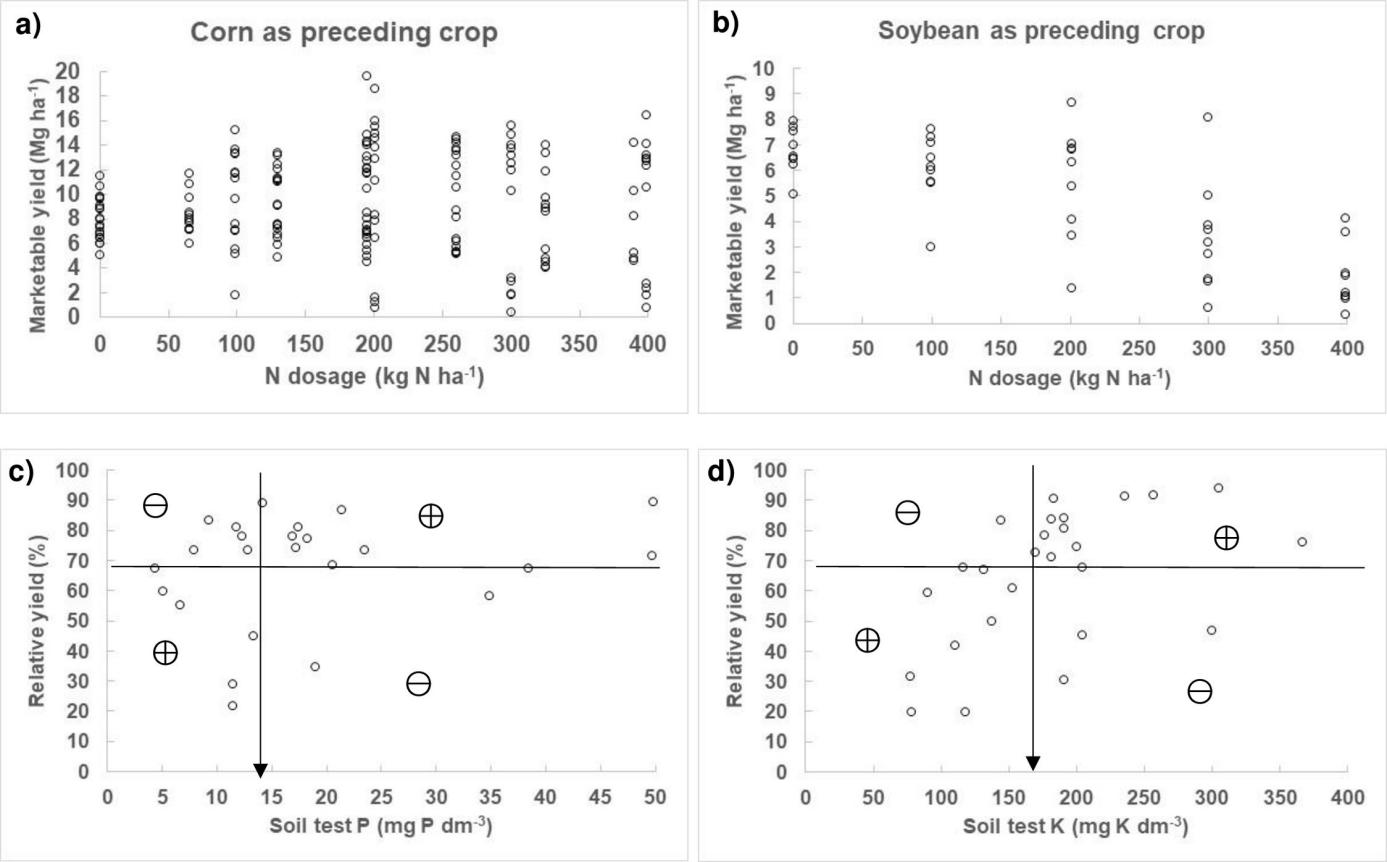
Response of garlic marketable yield, N dosage and soil tests for P and K recorded in experimental sites (2015–2017). The N model showed the apparent impact of preceding crops on yield and response to N fertilization (a, b). The P model indicated critical soil test P value, at classification accuracy of 0.708 (c). Classification accuracy of the K model was 0.815 (number of points in the ⊕ quandrants divided by total number of points) about soil test K close to 160 mg Mehlich-1 K dm^-3^ (d).

The association between garlic yield and N dosage in the experimental data set was different between corn and soybean (Fig 1). Maximum marketable garlic yield reached 20 Mg ha^-1^ after corn in comparison to 9 Mg ha^-1^ after soybean. However, soybean was only used a preceding crop in 2015, when climatic conditions were adverse due to excessive rainfall. The association between RY and soil test P showed classification accuracy of 0.708. Critical soil test P was ≈14 mg P dm^-3^ (Fig 1); this number is close to the separation between low and medium soil test P (CQFS RS/SC, 2016). The association between RY and soil test K was partitioned at ≈160 mg K dm^-3^, at classification accuracy of 0.815 (Fig 1); this number was close to the separation values (135–180 mg K dm^-3^) between medium and high soil test K.

### Machine learning models

**Fig 2:**
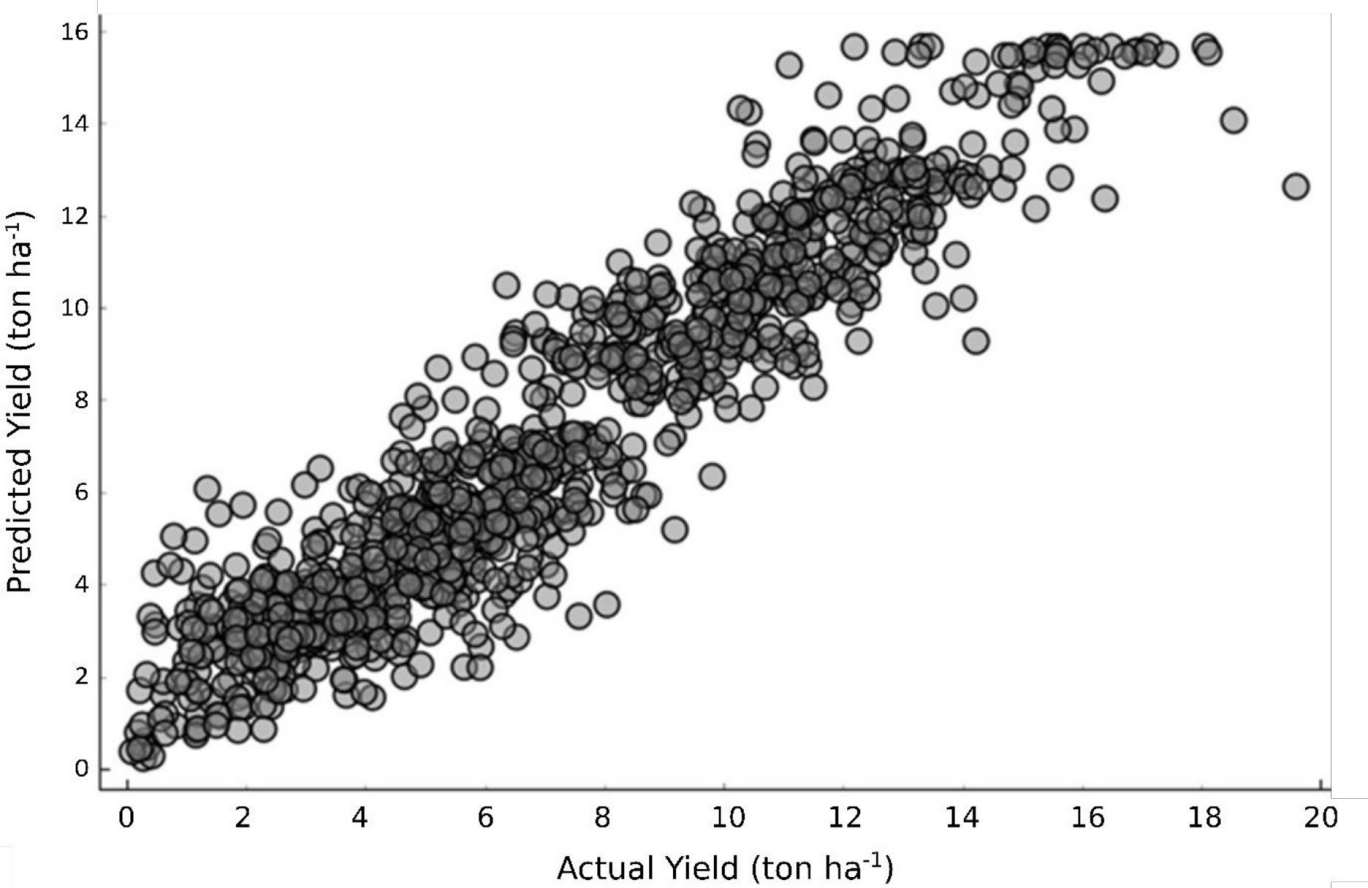
Random forest machine learning model for garlic yield prediction.

Random Forest, Gradient Boosting and Adaboost were the most performing ML regression models (Table 2). Because climate can be hardly used in predictions, and cultivar effect was negligible (data not shown), such features can be excluded to make predictions. Still, R^2^ values reached 0.832–0.882 when the top three ML models were used (e.g., Fig 2). Coefficient of variation (RMSE divided by mean) was 0.19 across the top ML models, for mean garlic yield of 7.3 ton ha^-1^. Random Forest also showed the highest model accuracy for the logistic partition between marketable and non-marketable yield (RMSE = 0.792; MAE = 0.615; R^2^ = 0.787).

**Table 2:** Accuracy of machine learning models across features or after excluding the cultivar and climatic indices (RMSE and MAE expressed as ton ha^-1^).

Learner	All features (Model 1)			Excluding cultivar and climatic indices (Model 2)		
	RMSE	MAE	R^2^	RMSE	MAE	R^2^
Random Forest	1.359	1.035	0.886	1.384	1.051	0.882
Adaboost	1.411	1.088	0.877	1.410	1.075	0.877
Gradient Boosting	1.403	1.095	0.879	1.653	1.226	0.832
Neural Network	1.605	1.221	0.841	2.032	1.514	0.745
KNN	1.889	1.429	0.780	3.123	2.478	0.399
Linear Regression	2.337	1.849	0.663	2.678	2.105	0.558

Fertilization treatments’ contribution to model accuracy was tested by fully, or sequentially, removing N, P and K fertilization from Model 2. R^2^ of Random Forest dropped to 0.797 after all fertilization treatments were removed from it. Nitrogen (N) was the most yield-impacting nutrient. R^2^ of Random Forest dropped from 0.882 to 0.812 after N fertilization effect was removed from the model. R^2^ values of Random Forest were little changed after P (0.873) or K (0.881) treatments were removed from the model, and this finding indicates the small effects of P and K fertilization on garlic yield.

### Model evaluation

**Fig 3:**
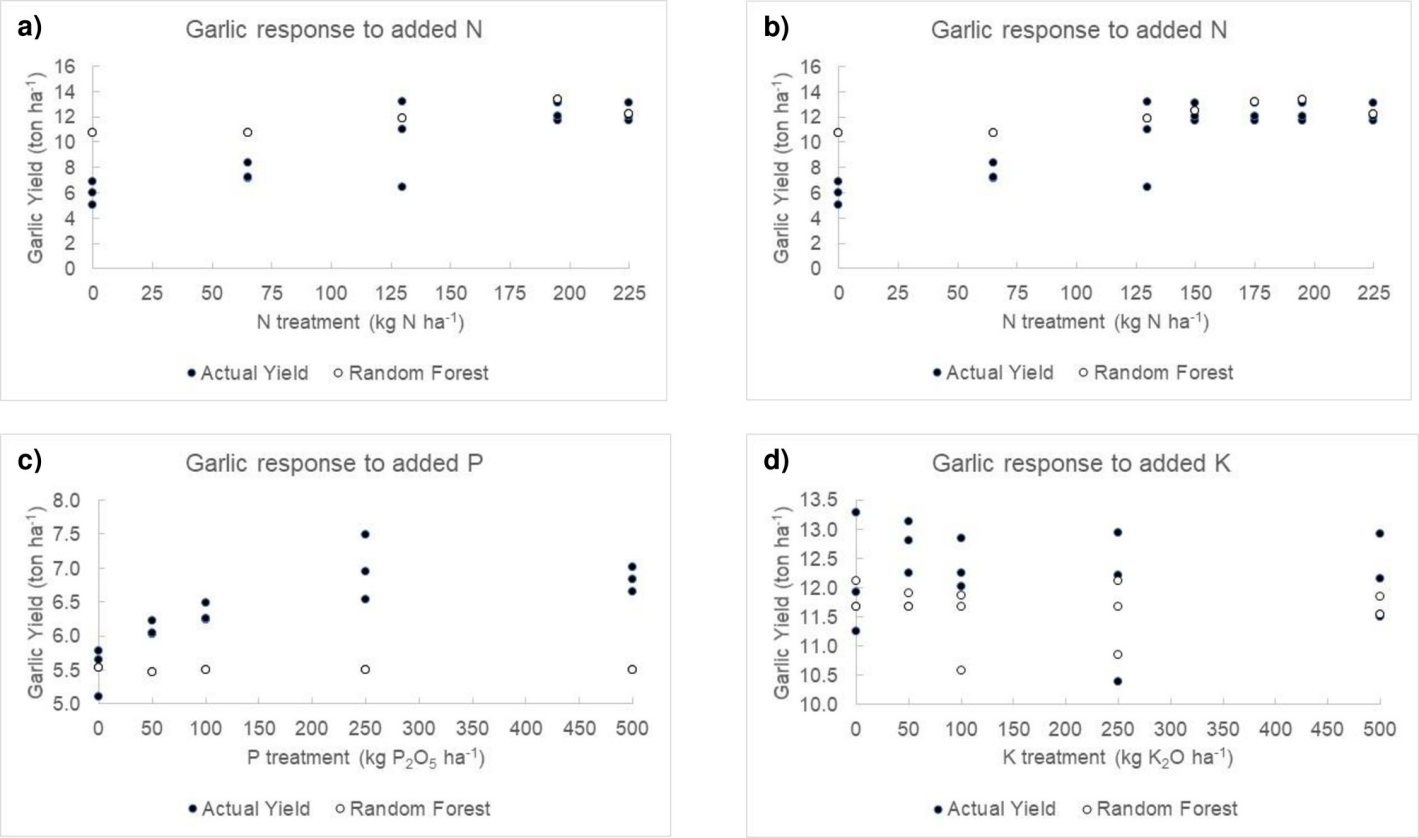
Garlic response to nutrient additions in N (2017) (a, b), P (2016) (c) and K (2016) (d) trials run to evaluate Random Forest predictions. In total, 150 and 175 kg N ha^-1^ were added to the N response model (b), in order to fill the gap between 130 and 195 kg N ha^-1^.

Garlic crops’ response to N, P and K fertilization treatments were predicted at three sites in 2016 and 2017 by using the calibration dataset. Models presented non-linear trajectory for N and stationary trajectories for P and K (Fig 3). Random Forest Model 2 predictions for garlic yield response to N addition were close to the observed yield. Based on model predictions for N, the dosage of 200 kg N ha^-1^ would be enough to maximize marketable yield across sites. The model did not predict response to K or P addition, despite apparent observed response, at 250 kg P_2_O_5_ ha^-1^.

## Discussion

### Early regional guidelines

The ‘universal’ state recommendations based on preceding crops and soil organic matter for N, as well as the association between RY and soil test P and K [55], proved to change in ML predictions. Nutrient recovery coefficients must vary due to water supply, soil test level, root competition in constrained rooting volume, and fertilizer dosing and placement [2, 59, 60]. The study by [26] challenged the belief that tropical soils’ ability to supply P mostly depends on crop recovery coefficients. Trials in tomato fields conducted in São Paulo State [26] showed that irrigated tomato plants responded much less to P addition than the predictions for it, which were based on crop removal and on an assumed P recovery coefficient. There was great potential to reduce costs with fertilization obtained with predictive models, without agronomic loss. Nevertheless, it is safe to compare the model’s predictions to state recommendations, or grower’s predictions to secure knowledge transfer.

### Model accuracy

Total N offtake ranged from 108 and 292 kg N ha^-1^ (avg. 207 kg N ha^-1^), P offtake ranged from 17 to 37 kg P ha^-1^ (avg. 29 kg P ha^-1^), and K offtake ranged from 67 to 167 kg K ha^-1^ (avg. 123 kg K ha^-1^) for garlic yields ranging from 9.1 to 24.2 Mg ton ha^-1^ (avg. 17.3 ton bulb ha^-1^) [6]. Mean garlic offtake was 12 kg N ton^-1^, 1.7 kg P ton^-1^ and 7.1 kg K ton^-1^.

Nitrogen (N) requirements were found to be much lower (≈200 kg N ha^-1^) in case study (Fig 3), than the ‘universal’ state recommendations of 300 kg N ha^-1^ [55]. For yield of 13 ton ha^-1^, crop N of take would be 156 kg N ha^-1^ (Fig 3). The chosen response curve, and range and number of doses, may also have impact on the optimum nutrient dosage [24, 25, 61]. Results presented by [52] showed total and marketable garlic yield non-linear response pattern under greenhouse conditions—it reached 320 kg N ha^-1^ at yield level of 9 Mg ha^-1^. Maximum marketable yield of 12.9–15.8 Mg ha^-1^, at 200 kg N ha^-1^, was recorded under field conditions in Mid-Eastern Santa Catarina State, where soil organic matter ranges from 3.9% to 4.9%, [14]. Mean fertilizer dosage of 222±30 kg N ha^-1^ was recorded in garlic farms, in Minas Gerais State, Brazil [8]. Nitrogen (N) applied by growers (2018–2019) ranged from 130 to 180 kg N ha^-1^ in the present collaborative study, and this finding reflects growers’ caution about the potential negative effects of N over-fertilization on crop quality. Optimum N dosage could be re-assessed at local scale by testing N treatments close to optimum experimentally derived N [24, 25]. This finding emphasizes the need of customizing fertilizer recommendations in order to reach factor-specific conditions, at local scale, save money to the grower, improve crop quality and reduce environmental footprint.

There is little information on garlic response to P and K addition under field conditions, in Brazil. Phosphorus (P) placement can increase garlic yield in low-P soils [60]. Significant yield difference between zero and 200 kg P_2_O_5_ ha^-1^ was reported in low-P soils (4.3 mg P-Mehlich-1 dm^-3^) recording total bulb yield of 11–14 Mg ha^-1^ [62]. On the other hand, a small yield response to 100–150 kg K_2_O ha^-1^ was reported by [63], when total yield levels were low (3.5 to 7 Mg ha^-1^) in high-K soil (47 mg K dm^-3^). Mean fertilizer dosage of 371±39 kg P ha^-1^, and 417± 62 kg K ha^-1^ in garlic farms, was recorded in Minas Gerais State, Brazil [8]. Growers (2018–2019) followed state recommendations for P and K, or over-fertilized soil—already showing very high test values. Hence, every grower made different decisions based on their own knowledge and perception about local conditions.

On the other hand, based on Random Forest results, it is recommended to significantly reduce N, P and K doses. Garlic can successfully grow even in low-P soils due to root colonization by arbuscular mycorrhizal fungi (AMF) [64, 65]. Plant Growth Promoting Rhizobacteria (PGPR) can also improve N and P supply to garlic in Southern Brazil soils [27]. Disregarding soil biological quality effects can have great impact on N and P prescriptions only based on soil test N and P, alone. Based on the “Biological Buffering Capacity—BBC”, crop yield and N uptake are closely linked to soil/crop interactions affected by the weather during the growing season, since plants are the path to transfer weather effects to changes in soil/crop interactions” [32]. However, BBC can be hardly measured under all combinations of factors and environmental conditions.

Very wet, dry or favorable climatic conditions were recorded during the 3-year experiment. Nonetheless, the data set did not cross over the numerous combinations of factors influencing garlic yield and quality in Southern Brazil. Acquiring more observational and experimental data from a larger number of predictors combinations, in collaboration with growers, could help updating ML models and improving nutrient management predictions Experimental and observational data can be turned into large datasets in order to support decision-making based on combining research results, growers’ results and knowledge, as well as to feed and run machine learning models to reach high crop yield based on the numerous combinations of site-specific factors [37].

## Conclusion

Based on the pilot study, ML models can be more accurate than state recommendations at local scale, as shown by the three case studies. Predictions have shown that growers can significantly reduce nutrient applications and yet reach potential marketable yield. However, ML models remain sensitive to the tested number of doses and to undocumented features in the data set, such as biological quality indices. Nevertheless, learners have the potential to support trustful technology transfer from research to the field, as well as to contribute to sustain the Brazilian garlic production. Customized fertilizer recommendations to site-specific conditions are promising tools to reach (i) high yield with economic viability; (ii) adequate plant mineral nutrition, (iii) high fertilizer-use efficiency. These achievements are possible by sharing larger and more diversified data sets among stakeholders.

## Supporting information

S1 File (DOCX)
