# Supplementary material for: Garlic (Allium sativum) feature-specific nutrient dosage based on using machine learning models
Source: PLoS One. 2022 May 17;17(5):e0268516. doi: 10.1371/journal.pone.0268516 (PMC9113611; doi:10.1371/journal.pone.0268516)
Supplement: S1 File — (DOCX) [file pone.0268516.s001.docx]

**S1 Table. Fertilizer treatments by cultivar in the experimental dataset (2015-2017).**

| Cultivars | #observations | Preceding crops | N | P | K |  |  |
| --- | --- | --- | --- | --- | --- | --- | --- |
|  |  |  | kg ha^-1^ | | |  |  |
| Chonan | 441 | Soybean, corn, bean, garlic, black oat | 0-400 | 0-349 | 0-667 |  |  |
| Ito | 450 | Soybean, corn, bean, garlic | 0-400 | 0-349 | 0-667 |  |  |
| Quitéria | 30 | Bean | 0-400 | 0-218 | 0-417 |  |  |
| Roxo Caxiense | 60 | Bean, garlic | 0-400 | 0-218 | 0-417 |  |  |
| São Valentim | 42 | Corn | 0-400 | 0-218 | 0-417 |  |  |

**S2 Table. Variation in soil properties in the Brazilian garlic data set**

| Property | Minimum | Median | Maximum |
| --- | --- | --- | --- |
| pH in water | 5.4 | 6.1 | 6.7 |
|  | cmol_c_ dm^-3^ | | |
| Cation Exchange Capacity | 12.7 | 14.0 | 22.6 |
| Ca | 6 | 8 | 18.9 |
| Mg | 1.1 | 2.6 | 5 |
|  | % | | |
| Clay | 39 | 60 | 70 |
| Soil Organic Matter | 2.9 | 3.8 | 5.7 |
|  | mg dm^-3^ | | |
| P | 3.4 | 18.5 | 158.6 |
| K | 60.2 | 254 | 650 |
| Zn‡ | 1.6 | 5.5 | 11.2 |
| Cu‡ | 2.7 | 6.9 | 12.5 |
| Mn‡ | 2.5 | 11.6 | 35 |
| Fe‡ | 1.3 | 1.9 | 5.1 |

‡ No data available in 2017 and 2019

**S3 Table. Variations in minimum and maximum values of climatic indices during the experimental period.**

| Climatic index | 2015 | 2016 | 2017 | 2018 | 2019 |
| --- | --- | --- | --- | --- | --- |
| Total rainfall (mm) | 966-1059† | 545-660 | 1272 | 455 | 396-431 |
| Degree-days (>5°C) | 1159-1795 | 1232-1396 | 1238 | 1298 | 1595-1629 |
| Length of the growing season (d) | 102-162 | 126-161 | 129 | 128 | 139-148 |

†Excess rainfall
